# Membrane Repair Deficit in Facioscapulohumeral Muscular Dystrophy

**DOI:** 10.3390/ijms21155575

**Published:** 2020-08-04

**Authors:** Adam J. Bittel, Sen Chandra Sreetama, Daniel C. Bittel, Adam Horn, James S. Novak, Toshifumi Yokota, Aiping Zhang, Rika Maruyama, Kenji Rowel Q. Lim, Jyoti K. Jaiswal, Yi-Wen Chen

**Affiliations:** 1 Research Center for Genetic Medicine, Children’s National Hospital, 111 Michigan Ave NW, Washington, DC 20010, USA; abittel@childrensnational.org (A.J.B.); sreetamasen@gmail.com (S.C.S.); dbittel@childrensnational.org (D.C.B.); AHorn@childrensnational.org (A.H.); jnovak@childrensnational.org (J.S.N.); AZhang@childrensnational.org (A.Z.); 2 Department of Genomics and Precision Medicine, The George Washington University School of Medicine and Health Science, 111 Michigan Ave NW, Washington, DC 20010, USA; 3 Department of Medical Genetics, University of Alberta, 116 St. & 85 Ave., Edmonton, AB T6G 2R3, Canada; toshifumi.yokota@ualberta.ca (T.Y.); yokotama@ualberta.ca (R.M.); kenjirow@ualberta.ca (K.R.Q.L.); 4 Department of Integrative Systems Biology, Institute for Biomedical Sciences, The George Washington University, 2121 I St. NW, Washington, DC 20052, USA

**Keywords:** FSHD, DUX4, membrane, repair, myoblast, myofiber, antisense oligonucleotide, MOE, gapmer, antioxidant, muscle

## Abstract

Deficits in plasma membrane repair have been identified in dysferlinopathy and Duchenne Muscular Dystrophy, and contribute to progressive myopathy. Although Facioscapulohumeral Muscular Dystrophy (FSHD) shares clinicopathological features with these muscular dystrophies, it is unknown if FSHD is characterized by plasma membrane repair deficits. Therefore, we exposed immortalized human FSHD myoblasts, immortalized myoblasts from unaffected siblings, and myofibers from a murine model of FSHD (*FLExDUX4*) to focal, pulsed laser ablation of the sarcolemma. Repair kinetics and success were determined from the accumulation of intracellular FM1-43 dye post-injury. We subsequently treated FSHD myoblasts with a *DUX4*-targeting antisense oligonucleotide (AON) to reduce *DUX4* expression, and with the antioxidant Trolox to determine the role of *DUX4* expression and oxidative stress in membrane repair. Compared to unaffected myoblasts, FSHD myoblasts demonstrate poor repair and a greater percentage of cells that failed to repair, which was mitigated by AON and Trolox treatments. Similar repair deficits were identified in *FLExDUX4* myofibers. This is the first study to identify plasma membrane repair deficits in myoblasts from individuals with FSHD, and in myofibers from a murine model of FSHD. Our results suggest that *DUX4* expression and oxidative stress may be important targets for future membrane-repair therapies.

## 1. Introduction

Facioscapulohumeral muscular dystrophy (FSHD) is an autosomal dominant neuromuscular disorder characterized by asymmetric muscle weakness, atrophy, fatty infiltration, and inflammation, typically beginning in the muscles of the face and periscapular region and progressing to the lower extremities over time [1]. In ~95% of cases, disease onset is linked to a contraction in the number of ~3.3 Kb, GC-rich D4Z4 repeats on chromosome 4q35 from 11–100 (average 25–35) in healthy individuals, to 1–11 repeats in FSHD1 [2,3]. This contraction leads to a loss of repressive histone marks, relaxation of chromatin, and DNA hypomethylation, which reverses the normal pattern of epigenetic repression of the D4Z4 locus and facilitates abnormal gene expression [3]. In approximately 5% of cases, individuals retain >10 D4Z4 repeats but demonstrate D4Z4 hypomethylation secondary to mutations in the gene SMCHD1 (structural maintenance of chromosomes flexible hinge domain containing 1) (FSHD2) [4]. Both forms of FSHD are characterized by aberrant expression of the double homeoprotein 4 (*DUX4*) transcription factor in the presence of a permissive 4qA haplotype (presence of a polyadenylation consensus sequence distal to the last D4Z4 repeat that stabilizes the *DUX4* transcript) [5]. *DUX4* activates a number of germline genes, immune mediators, apoptosis pathways, and alters RNA and protein metabolism, leading to progressive myopathy [6,7,8,9,10].

Current evidence suggests that repeated cycles of muscle damage, necrosis, and regeneration contribute to the development of fibrosis and intramuscular fat—common pathological features of FSHD that can contribute to impaired muscle performance [11,12]. However, in many patients with FSHD, plasma CK (a measure of muscle damage) levels are only slightly (<600 U/L) or moderately elevated (600–1500 U/L) [13,14,15] when measured at rest, and can be variable from day to day based on the individual’s level of physical activity [16,17]. The lower levels of plasma CK at rest indicate that FSHD is not characterized by baseline muscle fragility (as is found in Duchenne Muscular Dystrophy), but instead may demonstrate impaired responses to larger stresses—especially those that damage the sarcolemma. Muscle contraction can lead to sarcolemmal damage as force is transmitted from the sarcomere to the extracellular matrix [18]. Rapid repair of membrane disruption is essential to prevent myofiber apoptosis, and sarcolemmal repair deficits contribute to the skeletal muscle pathology in common muscular dystrophies, including dysferlinopathy [Limb girdle muscular dystrophy 2B (LGMD2B) and Miyoshi myopathy (MM)], and Duchenne Muscular Dystrophy (DMD, caused by dystrophin deficiency) [19,20]. Specifically, loss of spatio-temporal control of the signaling events required for membrane repair (e.g., rapid influx of calcium (Ca^2+)^, reactive oxygen species (ROS) production) in these muscular dystrophies compromises the removal of damaged cellular components and membrane re-sealing, thereby contributing to myofiber death and muscle atrophy [21,22]. Therefore, deficient membrane repair responses to muscle injury could also contribute to progressive myopathy in FSHD, but this has not been examined.

Chronic ROS-induced cell damage (oxidized DNA, lipid peroxides) is a central feature of FSHD that develops secondary to mitochondrial dysfunction (e.g., separation of inner and outer mitochondrial membrane, pathological swelling), and an insufficient antioxidant defense despite compensatory elevations in key antioxidant enzymes (due, in part, to the loss of antioxidative cofactors) [23,24,25,26,27]. While we previously demonstrated that carefully controlled mitochondrial ROS signaling is essential for muscle membrane repair, chronic oxidative stress (8–24 h) interferes with this process [28,29]. Additionally, recent chromosomal conformation capture and proteomic analyses revealed upregulation of membrane trafficking pathways (endocytosis and exocytosis) in FSHD myoblasts, suggesting that there may be imbalances in essential membrane repair pathways in FSHD [30,31].

Since FSHD muscle shares clinical features with other forms of muscular dystrophy that are associated with plasma membrane repair deficits and dysfunctional repair mechanisms, we hypothesized that FSHD muscles may also suffer from impaired plasma membrane repair capacity. Specifically, we hypothesized insufficient repair could be linked to *DUX4* mRNA expression and abnormal ROS levels in FSHD, and that addressing these defects may enhance repair. To test these hypotheses, we examined membrane repair in myoblasts from patients with FSHD and their unaffected siblings, as well as mature myofibers from a mouse model of FSHD. Further, we investigated the capacity for *DUX4* mRNA knockdown and antioxidant treatment to improve plasma membrane repair in these cells/tissues.

## 2. Results

### 2.1. Membrane Repair Deficit in FSHD Myoblasts

**Figure 1:**
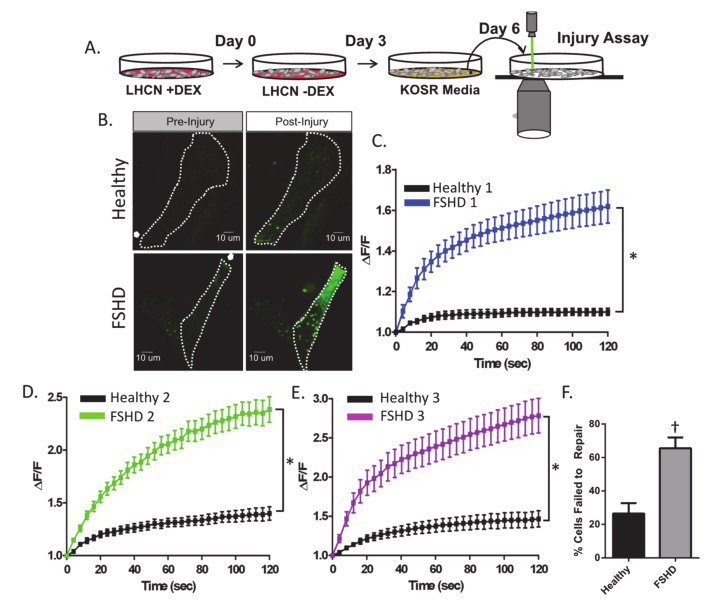
Immortalized myoblasts from individuals with Facioscapulohumeral Muscular Dystrophy (FSHD) display impaired membrane repair following laser ablation injury to the plasma membrane. (**A**) Outline of the cell culture protocol used in this study. Immortalized FSHD and unaffected myoblasts were cultured and passaged in growth media with dexamethasone (LHCN +DEX). For all experiments, cells were seeded onto gelatin-coated coverslips in 6-well dishes and cultured in growth media without dexamethasone (LHCN −DEX) for three days. After three days, cells were transitioned to 15% knockout serum replacement media (KOSR media) for three days before laser injury assay. (**B**) Images of FM1-43 dye entry (green) in injured myoblasts at baseline (pre-injury) and at 120 s post-injury. Arrow indicates location of laser injury. Dotted line indicates cell outline. (**C**–**E**) Change in intracellular FM1-43 dye fluorescent intensity (ΔF/F) over time for FSHD myoblasts (FSHD1 *n* = 33 cells, FSHD2 *n* = 26 cells, FSHD3 *n* = 31 cells) and unaffected control myoblasts (Healthy1 *n* = 30 cells, Healthy2 *n* = 24 cells, Healthy3 *n* = 31 cells) respectively. Greater dye entry indicates worse repair. (**F**) The percentage of healthy and FSHD cells that failed to repair. Values are mean ± SEM. * indicates significant genotype (FSHD vs. Healthy)*time interaction for FM1-43 intensity at *p* < 0.001. † denotes difference between FSHD and healthy myoblasts, independent *t*-test, *p* < 0.05.

First, we examined plasma membrane repair capacity in immortalized human myoblasts isolated from three individuals with FSHD and their first-degree relatives (Table 1) [32,33]. We have previously described that culturing these FSHD patient cells in normal growth media (LHCN) in the presence of dexamethasone maintains low levels of *DUX4*, enabling stable maintenance of these cells for extended periods [34]. However, shifting these cells to modified media (containing knockout serum replacement, KOSR) without dexamethasone provides a culture condition that leads to a higher level of endogenous *DUX4* expression in FSHD cells [34]. Therefore, we sequentially switched the cultured patient cells from basal to the modified media with dexamethasone to increase endogenous *DUX4* expression prior to membrane repair assessment (Figure 1A). To monitor the plasma membrane repair ability of these cells, we used a pulsed laser ablation injury assay in which the cell’s plasma membrane is injured with a 10 ms pulse of a focused laser while incubating in cell impermeant FM1-43 dye [35]. This injury allows the FM1-43 dye to enter the cell upon membrane disruption, causing an increase in fluorescence intensity as FM1-43 binds to the internal membrane [35]. We found that all three FSHD patient myoblasts allowed significantly greater amounts of FM1-43 dye entry into the cytosol, indicating significantly worse repair ability when compared to their unaffected first-degree relatives (significant tx*time interaction *p* < 0.001 for all three cell lines, Figure 1B–D). This deficit corresponded with a significantly greater percentage of FSHD patient myoblasts failing to repair following injury (*p* < 0.05 for all three cell lines, Figure 1C–E).

### 2.2. Antisense Oligonucleotide Treatment Improves Membrane Repair

**Figure 2:**
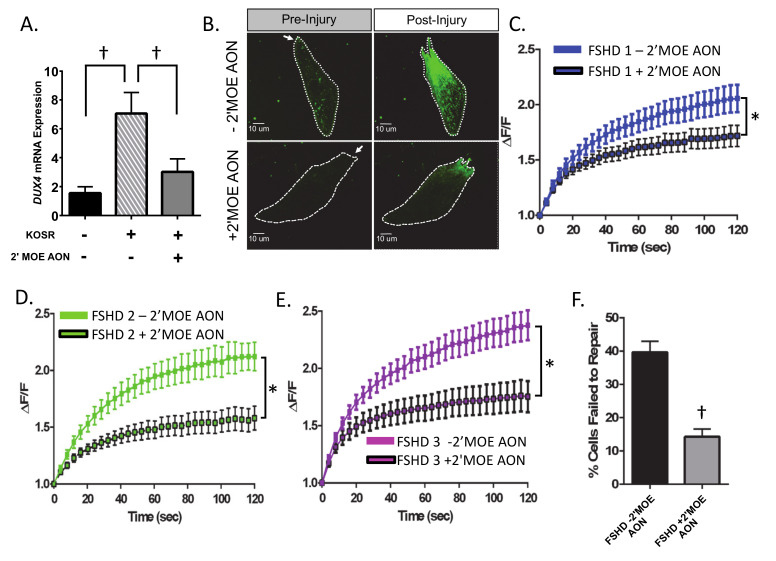
Treatment of FSHD-immortalized myoblasts with antisense oligonucleotides (2′-O-(2-Methoxyethyl)- oligoribonucleotide (2′MOE AON)) targeting *DUX4* improves membrane repair. (**A**) The culturing protocol (three days in LHCN −DEX, three days in 15% KOSR media) significantly increases *DUX4* mRNA expression in FSHD muscle cells. 2′MOE AON treatment reduces *DUX4* mRNA to baseline levels (*n* = 3 per cell line). (**B**) Images of FM1-43 dye entry in injured myoblasts at baseline (pre-injury) and at 120 s post-injury in control FSHD myoblasts, and FSHD myoblasts treated with 100 nM 2′MOE AON. Arrow indicates location of laser injury. (**C**–**E**) Change in intracellular FM1-43 dye fluorescent intensity (ΔF/F) over time for control FSHD myoblasts (FSHD1 *n* = 41 cells, FSHD2 *n* = 22 cells, FSHD3 *n* = 37 cells) and FSHD myoblasts treated with 100 nM 2′MOE AON (FSHD1 *n* = 39 cells, FSHD2 *n* = 22 cells, FSHD3 *n* = 33 cells). For all three cell lines, 2′MOE AON treatment significantly improves membrane repair (demonstrated by a significant reduction in dye entry post-injury) (*p* < 0.05). (**F**) Percentage of cells that failed to repair with and without 2′ MOE AON treatment across all three cell lines in each condition. Greater dye entry indicates worse repair. Values are mean ± SEM. * indicates significant treatment (+2′MOE AON vs. –2′MOE AON)*time interaction for FM1-43 intensity at *p* < 0.05. † denotes difference between FSHD myoblasts treated with and without 2′MOE AON, paired *t*-test, *p* < 0.05. KOSR = knock-out serum replacement.

Expression of the normally repressed transcription factor *DUX4* from the final D4Z4 repeat is believed to cause FSHD; and *DUX4* knockdown using phosphorodiamidate morpholino antisense oligonucleotides, short inhibitory siRNA, or RNA interference approaches reduces the FSHD atrophic phenotype in vitro and *DUX4*-induced myopathy in vivo [36,37]. Therefore, to determine if *DUX4* expression is also linked to the plasma membrane repair deficits in human FSHD myoblasts, we used a 2′-O-methoxyethyl (2′MOE) gapmer antisense oligonucleotide (AON) targeting exon 3 of the *DUX4* transcript to reduce *DUX4* mRNA. We treated immortalized FSHD myoblasts with the 2’MOE AON 48 h prior to plasma membrane injury and confirmed that this treatment reduced *DUX4* mRNA expression (Figure 2A, *p* < 0.05) such that there was no difference between *DUX4* expression in proliferating myoblasts (black bar in Figure 2A) and cells cultured in KOSR media following transfection with 2′MOE AON (gray bar in Figure 2A, *p* > 0.05). We then performed injury assays on AON-treated and untreated cells, as outlined above. We found that AON treatment significantly improved membrane repair capacity (reduced FM1-43 dye entry indicating improved repair relative to untreated immortalized myoblasts) in all three FSHD lines tested (*p* < 0.05, Figure 2B–E). These improvements corresponded to overall reductions in the percentage of cells that failed to repair (*p* < 0.05, Figure 2F). The data showed that reduction of *DUX4* mRNA level led to improvement of plasma membrane repair.

### 2.3. Antioxidant Treatment Improves Membrane Repair

**Figure 3:**
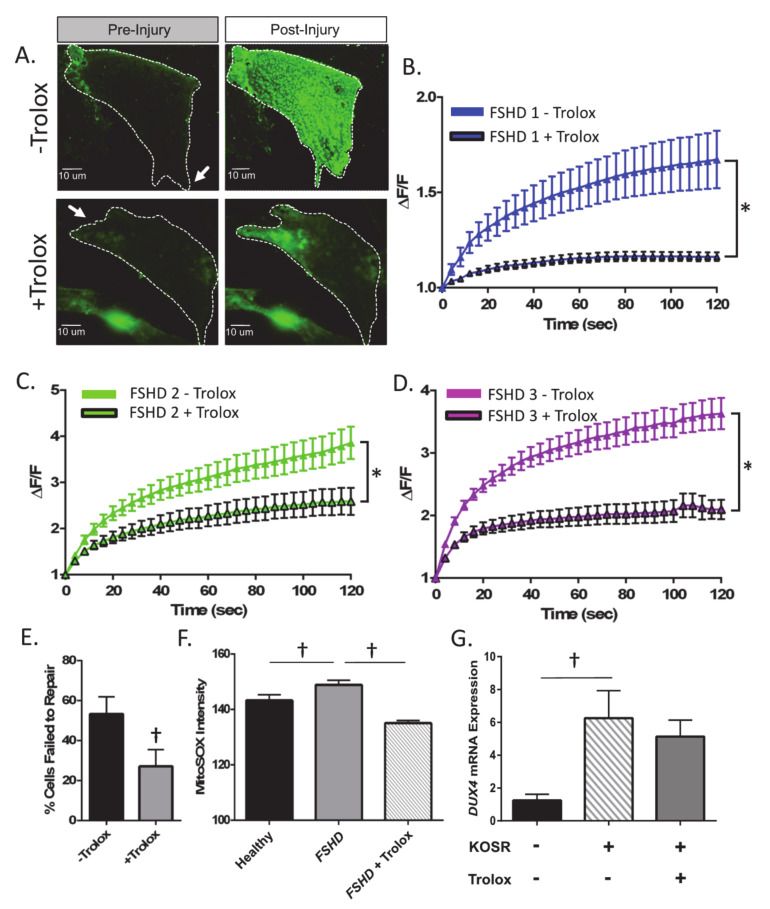
Treatment of FSHD-immortalized myoblasts with Trolox improves membrane repair. (**A**) Images of FM1-43 dye entry in injured myoblasts at baseline (pre-injury) and at 120 s post-injury in control FSHD myoblasts (FSHD1 *n* = 18 cells, FSHD2, *n* = 16 cells, FSHD3 *n* = 30 cells), and FSHD myoblasts treated for 24-h with 500 µM Trolox (FSHD1 *n* = 23 cells, FSHD2, *n* = 16 cells, FSHD3 *n* = 42 cells). Arrow indicates location of laser injury. (**B**–**D**) Change in intracellular FM1-43 dye fluorescent intensity (ΔF/F) over time for control FSHD myoblasts and FSHD myoblasts treated with Trolox. Greater dye entry indicates worse repair. (**E**) Percentage of cells that failed to repair with and without Trolox treatment. Values are mean ± SEM. (**F**) FSHD myoblasts (FSHD1) demonstrate elevated mitochondrial ROS, which was mitigated following 24 h of Trolox treatment (*n* = 32–37 cells/condition, *p* < 0.05). (**G**) The culturing protocol (three days in LHCN −DEX, three days in 15% KOSR media) significantly increases *DUX4* mRNA expression in FSHD muscle cells. However, 24-h treatment with 500 µM Trolox treatment did not affect *DUX4* mRNA expression. Values are mean ± SEM. * indicates significant treatment (+Trolox vs. –Trolox)*time interaction for FM1-43 intensity at *p* < 0.05. † denotes difference between FSHD myoblasts treated with and without Trolox, paired *t*-test, *p* < 0.05.

**Figure 4:**
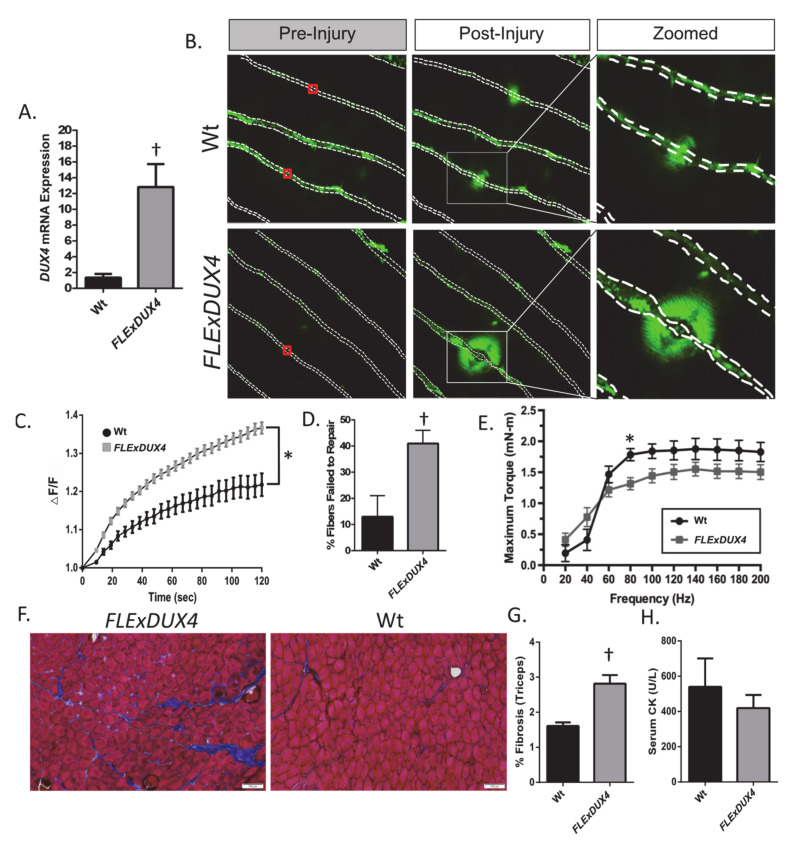
Mature myofibers from *FLExDUX4* mice demonstrate sarcolemmal repair deficit. (**A**) *DUX4* mRNA relative expression in *FLExDUX4* vs. Wt mice. (**B**) Representative images from membrane repair assays in *FLExDUX4* (*n* = 4) and wild type (Wt, *n* = 3) mouse biceps. Dotted lines denote the myofiber sarcolemma. Red box denotes site of injury. The extent of dye entry into the fiber (green dye in myofiber interior) is used to determine the kinetics of membrane resealing. (**C**) Mean ± SEM for change in fluorescence intensity of FM1-43 dye entry (ΔF/F) into the myofiber after laser injury in *FLExDUX4* and Wt mice. (**D**) Percentage of myofibers that failed to repair following membrane injury in *FLExDUX4* and Wt mice. (**E**) Force-frequency relationship curve for the tibialis anterior of 6.5-month old male *FLExDUX4* (*n* = 3) and Wt (*n* = 3) mice (significant interaction genotype*frequency, *p* < 0.05, with a significant difference identified at 80 Hz). (**F**) Masson’s Trichrome staining in the triceps of a *FLExDUX4* and a Wt mouse. (**G**) Percentage (of total area) of triceps fibrosis in 6.5 month-old male *FLExDUX4* (*n* = 5) and Wt (*n* = 3) mice. (**H**) Serum creatine kinase (CK) in *n* = 5 *FLExDUX4* (3 male, 2 female) and *n* = 5 Wt (3 male, 2 female) mice. * indicates significant genotype (*FLExDUX4* vs. Wt)*time interaction for FM1-43 intensity at *p* < 0.05. † denotes difference between *FLExDUX4* and Wt mice, independent *t*-test, *p* < 0.05.

Previous studies have found that oxidative stress compromises membrane repair and plays a central role in the pathophysiology of FSHD [25,27]. In vitro, treatment with antioxidants (e.g., retinoic acid) reduces ROS (via increased transcription of the ROS-scavenger glutathione peroxidase) and apoptosis in response to exogenous oxidative stress (H_2_O_2_ exposure) in human FSHD myoblasts, as well as *DUX4*-induced toxicity in C2C12 myoblasts, suggesting that antioxidant treatment could positively affect plasma membrane repair capacity [38,39]. To investigate if antioxidant treatment is beneficial for repair in FSHD myoblasts, we treated patient cells with Trolox (a water-soluble analog of vitamin E that serves as a ROS scavenger, membrane-associated antioxidant, and membrane-stabilizing agent in vivo) [40,41,42,43] and exposed them to the laser injury assay as described above. We found that 24 h of pre-treatment with Trolox significantly improved membrane repair capacity in all three FSHD-immortalized myoblast lines tested (*p* < 0.05, Figure 3A′D). Similar to treatment with 2′MOE AON, Trolox also reduced the percentage of cells that failed to repair across all three FSHD cell lines (*p* < 0.05, Figure 3E). Since Trolox has antioxidant effects, we also assessed myoblasts for levels of mitochondrial ROS using the mitochondrial superoxide indicator MitoSOX. We found that FSHD myoblasts demonstrate significantly elevated levels of mitochondrial ROS, which was mitigated by Trolox treatment (Figure 3F, *p* < 0.05). While previous studies have shown that antioxidants ameliorate *DUX4* toxicity, and that oxidative stress mediates *DUX4* expression secondary to activation of the DNA damage repair response, we did not identify any effect of Trolox treatment on *DUX4* mRNA expression (*p* > 0.05, Figure 4G) [23,44]. Taken together, these data suggest that antioxidant treatment may improve plasma membrane repair by reducing mitochondrial ROS levels in FSHD myoblasts independent of any effect on *DUX4* expression.

### 2.4. Membrane Repair Deficit in a Mouse Model of FSHD

The results of our previous work have established that the constitutive mechanisms of cell membrane repair, and the effectiveness of therapeutic approaches to improve repair capacity in myoblast models of skeletal muscle, are conserved in the case of myofibers [28,35,45,46,47]. Therefore, we sought to determine if mature FSHD skeletal muscle fibers display similar plasma membrane repair deficits to those observed in myoblast precursors. To this end, we tested the sarcolemmal repair capacity of myofibers from 4–6.5 month-old heterozygous male and female *FLExDUX4* mice, a well-established murine model of FSHD [48]. *FLExDUX4* mice express a human *DUX4*-fl transgene (including the 5′untranslated region, all three exons and both introns, and the endogenous polyadenylation signal) in a C57BL/6 background under the control of the Rosa26 promoter after cre-recombinase-mediated inversion [48]. In the absence of cre-recombinase, these mice were previously shown to present with low levels of “leaky,” skeletal muscle *DUX4* mRNA expression through antisense transcription that was consistent with the levels of *DUX4* mRNA measured in human FSHD myocytes [48]. We found similar evidence for leaky *DUX4* mRNA in our mice (Figure 4A, *p* < 0.05). Therefore, to determine if low levels of leaky *DUX4* affect sarcolemmal repair in mature muscle, we performed ex vivo laser injury assays on isolated whole biceps from *FLExDUX4* mice and their wild type (Wt) C57BL/6 littermates. The biceps were selected because the immortalized human myoblasts studied above were isolated from the biceps and deltoid of donors, and because the biceps are one of the most highly affected muscles in individuals with FSHD [49]. Consistent with myoblasts from the biceps/deltoid in human patients with FSHD, myofibers from *FLExDUX4* mice biceps demonstrated significantly worse repair than sex- and age-matched Wt littermates (Figure 4B,C, genotype*time interaction, *p* < 0.05), as well as a greater number of myofibers that failed to repair (Figure 4D, *p* < 0.05).

Since myofiber repair deficits were shown to contribute to muscle pathology in other muscular dystrophies [19,20,35], and because we identified repair deficits in *FLExDUX4* myofibers, we assessed for additional signs of muscular dystrophy in muscles shown to be highly affected in humans with FSHD (triceps, tibialis anterior (TA)) in the same cohort of *FLExDUX4* and Wt mice [49]. We observed a significant interaction between genotype and frequency for TA torque production (*p* < 0.05, a measure of muscle strength), with *FLExDUX4* mice demonstrating reduced torque production at 80 Hz (Figure 4E, *p* < 0.05) compared to their Wt littermates. We also fit a one-phase association growth model to the torque-frequency curve to determine the rate of rise in torque production as previously described [50]. This analysis revealed that *FLExDUX4* mice present with a reduced rate of rise in torque production (*FLExDUX4*: 0.016 ± 0.0005 vs. Wt: 0.019 ± 0.0006, *p* < 0.05, expressed in units of inverse frequency). We also identified elevated skeletal muscle fibrosis in the triceps (although overall fibrosis was limited to ~3%) (both *p* < 0.05, Figure 4F–G) in *FLExDUX4* mice compared to their Wt littermates. Serum creatine kinase, a measure of muscle damage or muscle membrane fragility, was not elevated in the *FLExDUX4* mice at rest (Figure 4H). These results indicate that *FLExDUX4* mice do not demonstrate signs of an unstable sarcolemma at rest (as is the case in other mouse models of muscular dystrophy such as the mdx mouse), but demonstrate deficits in repair following injury, which may contribute to muscle weakness and increased skeletal muscle fibrosis.

## 3. Discussion

In this study, we found that immortalized myoblasts from individuals with FSHD demonstrate impaired plasma membrane repair capacity compared to their healthy, unaffected first-degree relatives. Furthermore, intact myofibers from a transgenic mouse model of FSHD demonstrated similar sarcolemmal repair deficits compared to their wild type littermates. Based on previous research demonstrating the central role of *DUX4* and reduced ability of FSHD cells to handle increased oxidative stress [25,26], we showed that lowering *DUX4* expression with antisense oligonucleotides, and a membrane associating antioxidant/ROS-scavenger (Trolox), significantly improved repair in FSHD myoblasts. These findings demonstrate, for the first time, that membrane repair deficits may contribute to FSHD onset and/or progression, and that this deficit could be linked to *DUX4* expression and oxidative stress.

Similar to dysferlin-deficient LGMD2B and DMD, we identified that sarcolemmal repair deficits are a feature of the FSHD patient myoblasts and of skeletal muscle from a mouse model of FSHD (*FLExDUX4*) that presents with evidence of a slow myopathy (e.g., a significant increase in skeletal muscle fibrosis) [19,20,35]. Fatty infiltration and fibrosis are common features of FSHD skeletal muscle that may develop in response to repetitive cycles of muscle damage, regeneration, and expansion of fibroadipogenic progenitor pool [11,51]. However, with rather low *DUX4* expression in the absence of cre recombinase, *FLExDUX4* mice show no sign of spontaneous sarcolemmal fragility (no increase in serum creatine kinase level at rest), similar to humans with FSHD. Indeed, serum creatine kinase is usually only slightly or moderately elevated in patients when measured at rest, providing further evidence that FSHD may not be characterized by an unstable or fragile sarcolemma despite previous reports of sarcolemmal structural abnormalities identified in patient biopsies [13,14,15,52]. Instead, the deficits in sarcolemmal repair we identified may compromise recovery from muscle injury, or conditions in which muscle strain results in disruption of the plasma membrane [53]. In support of this hypothesis, previous reports suggest that muscle injury may trigger the insidious onset of symptoms in some individuals with FSHD [53]. These transient bouts of sarcolemmal injury, if not repaired properly, could contribute to progressive myopathy over time.

FSHD patients manifest many of the clinicopathological features common to LGMD2B and DMD, including lobulated (trabeculated) fibers with abnormal distributions of intramyofibrillar mitochondria, oxidative stress, and reductions in anti-oxidant defense mechanisms, abnormal calcium homeostasis, and mitochondrial dysfunction [19,20,54,55,56,57]. Unlike LGMD2B, MM, and DMD, FSHD is caused by gain-of-function mutation leading to expression of the transcription factor *DUX4*, which is known to have numerous detrimental downstream signaling effects in skeletal muscle [58]. To determine if *DUX4* expression was related to membrane repair deficits, we utilized a cell culture protocol that significantly increased endogenous *DUX4* expression in undifferentiated FSHD myoblasts [34]. Treatment with 2′MOE AON effectively reduced *DUX4* mRNA content by ~36%, and significantly improved membrane repair capacity by ~16%, suggesting that *DUX4* expression may be linked to membrane repair deficits. This hypothesis is further supported by the identification of *FLExDUX4* (a mouse model defined by leaky *DUX4* expression) myofiber repair deficits [48].

Repair deficits in LGMD2B have been linked to increases in the expression of vesicular trafficking pathway proteins not normally observed in muscle (e.g., synaptotagmin-like protein Slp2a/SYTL2 and the small GTPase Rab27A) [59]. Activation of an alternative exocytic pathway in LGMD2B could provide a compensatory vesicular trafficking mechanism to promote membrane repair in the absence of dysferlin [59]. A recent proteomic analysis of myoblasts expressing a *DUX4* transgene demonstrated that *DUX4* expression increases the levels of proteins involved in exocytosis—a central mechanism that contributes to membrane repair [30]. Increases in exocytic protein expression in FSHD myoblasts could, similarly, constitute a compensatory mechanism to overcome inherent repair deficits, which is worth further investigation.

Previous studies have also found impaired cell stress (e.g., unfolded protein and double stranded RNA stress) responses in myoblasts expressing *DUX4* that may contribute to muscle pathology [30]. Oxidative stress in FSHD develops in response to mitochondrial dysfunction and pro-oxidative stress alterations in the transcriptome, combined with an insufficient antioxidant response that develops despite elevations in antioxidant protein levels [25,60,61]. Oxidative stress has been linked to impairments in myoblast membrane healing, contractile dysfunction, apoptosis, atrophy, inflammation, and fatty infiltration, common symptoms of FSHD skeletal muscle [62,63,64,65,66]. While we did not identify a significant difference in peak torque production (with the exception of stimulation 80 Hz), the shape of the torque-frequency curve was altered in *FLExDUX4* muscle, characterized by a slower rate of rise in torque with increasing frequency. The rate of rise in torque is related to key muscle contractile properties (e.g., tetanic half-rise time), and similar abnormalities have been identified in other murine models of FSHD, as well as in humans [67,68]. Of note, ROS-induced oxidation of contractile proteins has been shown to reduce skeletal muscle force production and reduced calcium sensitivity, which could contribute to the observed declines in muscle function in FSHD [69]. Furthermore, while localized mitochondrial ROS are key signaling molecules required for successful membrane repair, chronic (or excessive) increases in ROS facilitate oxidative stress, which has been shown to compromise membrane repair capacity in C2C12 myoblasts [28,29]. Therefore, aberrant oxidative stress responses in FSHD may inhibit the required regulation of ROS signaling cascades needed for successful repair.

Based on this previous literature, we treated our FSHD myoblasts with the antioxidant Trolox—a, water-soluble analog of Vitamin E. Trolox maintains membrane stability and limits lipid peroxidation by neutralizing peroxyl radical (ROO•), singlet oxygen and superoxide anions, and direct intracellular ROS scavenging without changing antioxidant gene expression [40,41,42,43]. We found that treatment with Trolox for 24-h pre-injury significantly improved membrane repair capacity in all three FSHD cell lines tested while reducing levels of mitochondrial ROS (measured with the superoxide indicator MitoSOX). Our findings coincide closely with those of Howard et al. (2011), who reported that 24-h treatment with Trolox significantly improved membrane repair capacity in HeLa and BS-C-1 cells, as well as mouse myocytes by reducing oxidative stress during an H_2_O_2_ challenge [70]. These results are also consistent with our recent demonstration that another membrane-stabilizing compound known to prevent oxidation-induced lipid peroxidation improves sarcolemmal repair ability in LGMD2B patients and mouse models [46]. However, Trolox treatment did not affect DUX4 expression, suggesting that lipid peroxidation may not augment *DUX4* expression in these cells. Taken together, these findings suggest that antioxidants may improve membrane repair deficit in FSHD myoblasts. Further research into the mechanisms of improved membrane repair following Trolox treatment in FSHD myoblasts is needed to determine if lipid-directed antioxidants are required, or if general cellular antioxidants provide the same benefit. Presserieux et al. (2015) found that dietary supplementation with vitamin E, zinc, vitamin C, and selenium for 17 weeks increased quadriceps strength and endurance, further highlighting a potential link between membrane repair and muscle function [71].

Finally, a major challenge in understanding the effects of *DUX4* expression on skeletal muscle is the sporadic and rare nature of *DUX4* expression in vivo and in vitro. Indeed, previous studies have estimated that the expression of *DUX4* occurs in as few as 1/1000 nuclei in cultured FSHD myoblasts [72]. Single-cell RNAseq studies have reported *DUX4* expression in a small percentage of FSHD1 and FSHD2 myoblasts with as few as 1/217 cells demonstrating an FSHD transcriptional signature (expressing >5 *DUX4* biomarkers per cell) [73,74]. However, we identified deficits in membrane repair in the majority of myoblasts we tested from all three FSHD donors, with ~60–80% failing to repair based on the kinetics of FM1-43 dye entry after injury. This may be due to the known nature of *DUX4* expression, which occurs in a burst-like manner, leading to a wide range of detrimental downstream transcriptomic and epigenetic changes that create a cellular memory of *DUX4* expression [10]. These changes could result in morphological abnormalities that may persist even as gene expression returns to baseline, including accumulation of oxidative damage and mitochondrial dysfunction that could inhibit successful membrane repair. Over time, repeated injury and accumulated *DUX4* signaling could negatively affect muscle health and contribute to the onset or progression of apoptosis, atrophy, and fibrosis. If so, membrane repair capacity could be an important target for future therapeutic development.

## 4. Materials and Methods

### 4.1. Immortalized Human Myoblasts

Immortalized myoblasts (from three separate individuals with FSHD, and from their healthy, first-degree relatives) were obtained from Dr. Woodring Erik Wright at UT Southwestern University (Texas, USA) and the Senator Paul Wellstone Muscular Dystrophy Cooperative Research Center at Boston Biomedical Research Institute (Boston, MA, USA). Initial tissue biopsies were collected from the biceps and deltoid muscles [32]. The isolation, immortalization, and molecular diagnosis of these primary myoblasts have been described previously [32,33]. The characteristics of the donors are described in Table 1.

### 4.2. Cell Culture

Culturing conditions were modified from Pandey et. al. (2015) [34]. Briefly, the cells were grown in a 25 cm^2^ flask which was coated with 0.1% gelatin (Sigma-Aldrich, St. Louis, MO, USA). The cells were cultured in growth media consisting of Dulbecco’s modified Eagle’s medium (DMEM) and media 199 (Life Technologies, Carlsbad, CA, USA) in 4:1 ratio, with 0.8 mM sodium pyruvate (Life Technologies), 3.4 g/L sodium bicarbonate (Sigma-Aldrich), 15% fetal bovine serum (Thermo Fisher Scientific, Waltham, MA, USA), 0.03 µg/mL zinc sulfate (Thermo Fisher Scientific), 1.4 µg/mL vitamin B12 (Sigma-Aldrich), 0.055 µg/mL of dexamethasone (Sigma-Aldrich), 2.5 ng/mL recombinant human hepatocyte growth factor (MilliporeSigma, Burlington, MA, USA), 10 ng/mL basic fibroblast growth factor (Biopioneer, San Diego, CA, USA), and 10% 0.02 M HEPES (Life Technologies) at 37 °C, 5% CO_2._ The addition of dexamethasone suppresses DUX4 expression to facilitate cell proliferation, and removal of dexamethasone permits DUX4 expression at the level of the human donor from which they were derived [34]. Cells were passaged when around 70% confluent with phosphate buffered saline without calcium and magnesium (PBS) (Life Technologies) and TrypLE (Invitrogen, Carlsbad, CA, USA).

For all experiments, proliferating immortalized myoblasts were seeded in 6-well culture dishes at a density of 5.0 × 104 cells/well. Each well contained a coverslip coated with 0.4% gelatin (Sigma-Aldrich). For three days cells were allowed to proliferate in growth media without dexamethasone. After the cells reached 30% confluence, the cells were cultured in 15% KOSR (serum replacement) with Dulbecco’s modified Eagle’s medium (DMEM) and media 199 (Life Technologies) in 4:1 ratio, 2 mM L-glutamine (Life Technologies), 1 mM sodium pyruvate (Life Technologies), and 0.02 M HEPES (Life Technologies). After 3 days in KOSR media, the coverslips were removed from the wells and used for membrane repair assays. We previously reported that these culture conditions stimulate *DUX4* expression in the immortalized FSHD myoblasts used in this study [34].

### 4.3. Antisense Oligonucleotide and Antioxidant Treatments

During the transition to KOSR media, cells were transfected with a 100 nM 2′-O-(2-Methoxyethyl)-oligoribonucleotide (2′MOE) gapmer targeting the *DUX4* mRNA using Lipofectamine RNAi Max reagent (Life Technologies) per manufacturer’s instructions. The sequence for the 2′MOE gapmer targeting *DUX4* exon 3 was as follows: 5′**CCTAG**ACAGCGTCGG**AAGGT**3′ containing a fully phosphorothioated backbone with 2′MOE modified tails (bold type). After 6 h, cells were washed twice with PBS and transferred to 15% KOSR media for two days before imaging. For antioxidant assays, cells were incubated for 24 h with 500µM Trolox [70] in 15% KOSR media prior to imaging. 

### 4.4. Live Cell Imaging & Laser Injury Assays

The focused laser injury assay was adapted from the protocol used by Defour et. al. (2014) [35]. Briefly, cells cultured on coverslips were washed three times with PBS and transferred to cell imaging media (CIM; Hanks Balanced Salts Solution with 10 mM HEPES, 2 mM Ca^2+^, pH 7.4) containing 1 mg/mL of the cell impermeant FM1-43 dye, and then placed in a Tokai Hit microscopy stage-top ZILCS incubator (Tokai Hit Co., Fujinomiya-shi, Japan) maintained at 37 °C. For laser injury, a 1–2 mm^2^ area was irradiated for 10 ms with a pulsed laser (Ablate!, 3i Intelligent Imaging Innovations, Inc. Denver, CO, USA). Cells were imaged using an inverted IX81 Olympus microscope (Olympus America, Center Valley, PA, USA) custom-equipped with a CSUX1 spinning disc confocal unit (Yokogawa Electric Corp., Tokyo, Japan). Images were acquired using Evolve 512 EMCCD (Photometrics, Tucson, AZ, USA) at 2 Hz. Image acquisition and laser injury was controlled using Slidebook 6.0 (Intelligent Imaging Innovations, Inc., Denver, CO, USA). The change in FM1-43 dye fluorescence intensity (ΔF/F0) during the course of the imaging (120 s) was averaged for each condition and plotted. Successful repair was determined by the entry of FM1-43 dye into the cell interior, where a plateau in FM1-43 dye increase indicated successful repair, and failure to repair was indicated by unabated FM1-43 dye increase. To detect mitochondrial ROS, cells were incubated in 2.5 μM mitoSOX (Molecular Probes, Eugene, OR, USA) for 10 min at 37 °C per the manufacturer’s instructions. After washing with pre-warmed CIM, cells were imaged on the IX81 Olympus microscope using 510/580 nm excitation/emission filters.

### 4.5. Mouse Myofiber Injury

The animal procedures were approved by the IACUC (Protocol No. 00030340) at the Children’s Research Institute at the Children’s National Hospital. For all animal studies we used mature, adult mice between 4–6.5 months of age. For myofiber laser injury, four B6(Cg)-Gt(ROSA)26Sortm1.1(DUX4*)Plj/J (*FLExDUX4*) heterozygous mice (3 male, 1 female), and three of their C57BL/6 wild type littermates (2 female, 1 male) were euthanized by CO_2_ asphyxiation and cervical dislocation [37]. Whole biceps muscles were immediately dissected and mounted in pre-warmed Tyrode’s buffer (119 mM NaCl, 5 mM KCl, 25 mM HEPES buffer, 2 mM CaCl_2_, 2 mM MgCl_2_, 6 g/L glucose, pH 7.4) containing 1 mg/mL of FM1-43 dye, and injured and imaged as described above for myoblasts. The biceps muscle was studied because the immortalized myoblasts were isolated from the biceps and deltoid of human donors, and because the biceps is one of the primary muscles affected in FSHD [49]. Repair kinetics were determined based on the accumulation of FM1-43 dye in the myofiber cytosol. 

### 4.6. Mouse Skeletal Muscle Fibrosis Accumulation

To measure accumulation of intrafascicular connective tissue, 6 Wt (3 male, 3 female), and 10 *FLExDUX4 (*5 male, 5 female*)* mice were euthanized by CO_2_ asphyxiation and cervical dislocation. The triceps muscle was studied because it is one of the primary muscles affected in humans with FSHD. Samples were immediately dissected, mounted on cork with tragacanth gum, flash-frozen in liquid nitrogen-chilled isopentane, and stored at −80 °C. 8 µm thick sections were cut from the triceps using a Leica CM1950 cryostat microtome (Leica Microsystems, Buffalo Grove, IL, USA). Connective tissue was quantified from muscle cross sections using Gomori Trichrome staining (Abcam Trichrome Stain Kit, Abcam, Cambridge, MA, USA).

### 4.7. Mouse Skeletal Muscle Torque Production

To measure in-vivo torque production of the anterior crural muscles (tibialis anterior, extensor digitorum longus, peroneus tertius, and extensor hallucis longus), 3 male Wt and 3 *FLExDUX4* mice were anesthetized with 1.5% isoflurane-mixed oxygen, hair was removed from the left lower hind limb, and the foot was attached to a servomotor for torque measurement (Aurora Scientific, Aurora Canada). Dorsiflexion was tested because muscles of the anterior compartment are some of the most highly affected muscles in humans with FSHD [49]. Muscle contraction was stimulated using Pt-Ir needle electrodes inserted percutaneously adjacent to the peroneal nerve using 1 to 2 mA (stimulator model 701C, Aurora Scientific). Peak isometric torque was measured in response to 20, 40, 60, 80, 100, 120, 140, 160, 180, and 200 Hz. The rate of rise in torque was modeled using the exponential equation T = C × (1-e^−Df^), where T = torque produced at the given frequency (f), C = maximal torque, D = the rate of rise in torque, and f = frequency [50].

### 4.8. Mouse Serum Creatine Kinase Assay

Blood was collected in Eppendorf tubes from five Wt (3 male, 2 female) and five *FLExDUX4* (3 male, 2 female) mice after CO_2_ asphyxiation and cervical dislocation. Tubes were centrifuged at 22,000 rpm for 15 min. Following centrifugation, the supernatant was transferred to a new tube. Serum creatine kinase was analyzed by the University of Missouri Veterinary Medicine Diagnostic Lab.

### 4.9. Quantitative RT-PCR

For immortalized myoblasts, total RNA was purified by adding Trizol reagent directly to the cells after being washed twice in PBS, in accordance with the manufacturer’s instructions (Invitrogen). For mouse skeletal muscle, the tibialis anterior (not previously tested during torque measurements) from seven (4 female, 3 male) *FLExDUX4* and eight (4 male, 4 female) Wt mice were homogenized under liquid nitrogen by mortar and pestle. The fine powder was transferred to a 1.5 mL Eppendorf tube with a spatula and mixed with trizol reagent for RNA extraction. RNA was separated with chloroform, precipitated with isopropanol, and washed with 75% ethanol. Genomic DNA was removed and RNA was purified using the Qiagen RNeasy Plus Mini Kit (Qiagen, Hilden, Germany). For both cells and muscle, first strand cDNA was reverse-transcribed from 2 µg total RNA using Superscript IV (Life Technologies) and oligo dT primers. The cDNA was amplified in triplicate in SYBR Green PCR Master Mix (Life Technologies) using 1 µL cDNA template (20 ng cDNA) and 200 µM of forward and reverse primers in a total reaction volume of 20 µl. The thermal cycling conditions included 50 °C for 2 min, 95 °C for 10 min, followed by 40 cycles of amplification using the condition of 95 °C for 15 s then 60 °C for 1 min. The primer sequences used were: *DUX4*: (Forward) 5′ CCCAGGTACCAGCAGACC3′, (Reverse) 5′TCCAGGAGATGTAACTCTAATCCA3′; human *GAPDH* (Forward) 5′ TTGTCAAGCTCATTTCCTGGTATG3′, (Reverse) 5′ GTGAGGGTCTCTCTCTTCGACTTGT3′; mouse GAPDH (Forward) 5′ TTGTCAGCAATGCATCCTGC3′, (Reverse) 5′CCGTTCAGCTCTGGGATGAC3′. Relative gene expression was analyzed using the delta delta ct method.

### 4.10. Statistical Analysis

Differences in membrane repair kinetics (FM1-43 dye entry) were identified using mixed between-within repeated measures ANOVAs with genotype (FSHD vs. Healthy, *FLExDUX4* vs. Wt) or treatment (2′MOE AON vs. no 2′MOE AON, Trolox vs. no Trolox) entered as a between-subjects factor, and time entered as a repeated within-subjects factor. Differences in peak torque in the tibialis anterior were also determined with a mixed between-within repeated measures ANOVA with genotype (*FLExDUX4* vs. Wt) entered as a between-subjects factor, and frequency entered as a repeated within-subjects measure. Independent *t*-tests were used to identify differences in the percentage of cells that failed to repair between FSHD and healthy cells, the percentage of cells that failed to repair between 2′MOE AON and Trolox treatment conditions, serum creatine kinase, rate of rise in torque production, and skeletal muscle fibrosis. One-way ANOVAs were used to identify differences in cellular ROS, and *DUX4* mRNA expression following AON and Trolox treatments. The Benjamini–Hochberg procedure was used to control for multiple comparisons for all repeated measures. An alpha level of 0.05 was used for significance. All analyses were completed in SPSS version 25 (IBM, Amronk, NY, USA).

## 5. Conclusions

This study is the first to identify plasma membrane repair deficits in FSHD patient-derived myoblasts, and in the skeletal muscle of a murine model of FSHD that recapitulates the key features of the human disease. Based on the observed improvements in repair following *DUX4* mRNA knockdown and antioxidant treatment, our results also suggest that repair deficits may be linked to *DUX4* expression and oxidative stress. Deficient membrane repair represents a novel potential disease mechanism that may contribute to skeletal muscle myopathy in FSHD. Further study is needed to determine if human myofibers demonstrate sarcolemmal repair deficits, and to clarify the potential role of *DUX4* and oxidative stress on membrane repair in FSHD.

## Figures and Tables

**Table 1:** Clinical Characteristics of Muscle Biopsy Donors. Information from Homma et al. (2012) [32].

Donor	Familial Relationship	Gender	Age	*EcoRI/BlnI Fragment Sizes (kb)*	Deltoid Strength	Biceps Strength
FSHD1	Proband	M	42	18, >48	4+/5	4+/5
Healthy1	Brother of FSHD1	M	46	>48, >48	5/5	5/5
FSHD2	Proband	M	67	28, >112	5/5	4+/5
Healthy 2	Brother of FSHD2	F	60	107, >112	5/5	5/5
FSHD3	Proband	F	56	20, 97	5-/5	4+/5
Healthy 3	Sister of FSHD3	F	60	59, 93, 97	5/5	5/5

FSHD diagnosis was confirmed by the presence of muscle weakness, and ExoRI/BlnI restriction fragment <35 Kb, and a 4qA telomere allele. Muscle strength was graded according to the Medical Research Council (MRC) scale where 5/5 indicates full strength. FSHD = Facioscapulohumeral Muscular Dystrophy, M = male, F = female.

## Abbreviations

AON	Antisense Oligonucleotide
CK	Creatine Kinase
DEX	Dexamethasone
DMD	Duchenne Muscular Dystrophy
FSHD	Facioscapulohumeral Muscular Dystrophy
KOSR	Knockout Serum Replacement
LGMD2B	Limb Girdle Muscular Dystrophy 2B
MM	Miyoshi Myopathy
ROO•	Peroxyl Radical
ROS	Reactive Oxygen Species
Slp2a/SYTL2	Synaptotagmin-like protein
TA	Tibialis Anterior
Wt	Wild type
2′MOE	2′-O-(2-Methoxyethyl)-oligoribonucleotide
